# Impact of Meal Timing and Chronotype on Food Reward and Appetite Control in Young Adults

**DOI:** 10.3390/nu12051506

**Published:** 2020-05-22

**Authors:** Kristine Beaulieu, Pauline Oustric, Shaea Alkahtani, Maha Alhussain, Hanne Pedersen, Jonas Salling Quist, Kristine Færch, Graham Finlayson

**Affiliations:** 1 School of Psychology, University of Leeds, Leeds LS2 9JT, UK; k.beaulieu@leeds.ac.uk (K.B.); pspjo@leeds.ac.uk (P.O.); 2 Department of Exercise Physiology, College of Sport Sciences and Physical Activity, King Saud University, Riyadh 11451, Saudi Arabia; shalkahtani@ksu.edu.sa; 3 Department of Food Science and Nutrition, College of Food and Agricultural Sciences, King Saud University, Riyadh 11451, Saudi Arabia; mhussien@ksu.edu.sa; 4 Steno Diabetes Center Copenhagen, DK-2028 Gentofte, Denmark; hanne.pedersen.12@regionh.dk (H.P.); jonas.salling.quist@regionh.dk (J.S.Q.); kristine.faerch@regionh.dk (K.F.); 5 Department of Biomedical Sciences, University of Copenhagen, DK-2200 Copenhagen, Denmark

**Keywords:** chrono-nutrition, diurnal rhythms, meal timing, body composition, appetite, liking and wanting, satiety

## Abstract

Early meal timing and chronotype are associated with lower BMI, but their impact on appetite is poorly understood. We examined the impact of meal timing and chronotype on appetite and food reward. Forty-four adults were divided into early (EC; Morningness–Eveningness Questionnaire (MEQ) score = 55 ± 5) or late chronotype (LC; MEQ score = 40 ± 6) and assessed for body mass index, habitual energy intake (EI; three-day online dietary record) and eating behavior traits from the Three-Factor Eating Questionnaire (TFEQ). Participants attended the laboratory after ≥3 h fast on two occasions for early (AM; 8–10 a.m.) and late (PM; 4–6 p.m.) counterbalanced testing sessions in a 2 × 2 design. Appetite ratings and food reward (validated diurnal Leeds Food Preference Questionnaire) were measured in response to a standardized test meal. LC was associated with higher BMI (*p* = 0.01), but not with EI or TFEQ. The composite appetite score was lower in AM than PM (M_Δ_= −5 (95% CI −10, −0.2) mm, *p* = 0.040). Perceived test meal fillingness was higher in AM than PM and EC compared to LC (*p* ≤ 0.038). Liking and wanting high-fat food were lower in AM than PM (*p* ≤ 0.004). The late chronotype was associated with greater desire for high-fat food (*p* = 0.006). To conclude, early meal timing and early chronotype are independently associated with smaller appetite and lower desire for high-fat food.

## 1. Introduction

Human eating patterns are heterogeneous and erratic [1], and meal timing is variously influenced by socio-cultural [2] and hereditary [3] factors. An accumulating body of research indicates that the timing of food intake can influence energy balance and metabolism, with evening eating associated with greater energy intake and BMI compared to morning eating [4,5,6]. Indeed, scientific bodies now recognize that patterns of daily energy intake, including meal timing and frequency, influence the management of body weight and cardiometabolic risk [7]. Dietary interventions targeting food timing have been proposed for the treatment of obesity and obesity-related diseases, but evidence is limited [8]. Some studies have reported that greater food intake in the morning compared to the evening leads to more favorable weight loss outcomes, independent of total energy intake [9,10]. While the mechanisms for these effects are yet to be fully elucidated, they could include an altered metabolic response to meals [11,12], satiety [13], food choice and macronutrient composition [1,4] or other energy balance-related behaviors such as physical activity [12]. Indeed, observational evidence suggests the satiety value of food may be greater earlier in the day compared to later [13,14].

There is individual variability in diurnal rhythms for preferred timing of sleep, physical activity and eating, defined as chronotype. Chronotype differences can be measured through, for example, Horne and Ostberg’s Morningness–Eveningness Questionnaire [15]. Evening (late) chronotype is associated with higher BMI [16], binge eating behaviors [17] and greater evening energy intake [18], specifically from fewer fruits and vegetables [4], and more fast food and alcohol [19]. In contrast, morning (early) chronotype is positively associated with cognitive restraint, and inversely associated with disinhibition and susceptibility to hunger [20]. Interestingly, there is evidence suggesting that the relationship between meal timing and BMI is influenced by chronotype [16]. This suggests a potential interaction between meal timing and chronotype on appetite and eating behavior.

A very limited number of studies have examined diurnal or circadian variations in appetite under controlled conditions. Indeed, most of these studies have relied on self-reported food intake obtained from, e.g., food diaries. Measurement of appetite across the diurnal cycle is methodologically challenging as the appropriateness of components and size of the meals at different times of the day may influence outcomes. Therefore, there is a need to standardize the methodology across the day to assess diurnal variations in appetite and food reward.

Food reward includes the perception of liking food, but also involves other motivational drivers of food choice and intake, such as wanting [21]. Liking and wanting influence the strength of satiety, direction of food preferences and control over food intake [22]. They also play a role in expressions of overeating such as binge eating [23]. To our knowledge, however, no studies have assessed diurnal variations in food reward in humans. The Leeds Food Preference Questionnaire (LFPQ) methodology provides a framework, based on responses to an array of food images, to interpret the impact of interventions on liking and wanting as separate and distinct processes; however, an array of images that is equally suitable for different times of day has yet to be developed [24].

Therefore, the aim of this study was to examine whether meal timing and chronotype affect appetite and reward responses to food. Importantly, as a preliminary step, we sought to validate a test meal and LFPQ array of food images that were time-of-day appropriate for both early and late meal timing in young British adults.

## 2. Materials and Methods 

### 2.1. Development of Diurnal Leeds Food Preference Questionnaire and Test Meal

To increase the internal validity of the study design and outcomes (i.e., diurnal assessment of appetite and food preference), it was necessary to create an LFPQ array of food images (food categories of: high-fat savory, low-fat savory, high-fat sweet, and low-fat sweet) and a laboratory test meal that would be appropriate for early and late day consumption. In a preliminary study, we identified and photographed 28 foods (seven per food category) that two authors (P.O., G.F.) judged to be culturally and time-of-day appropriate for consumption both earlier and later in the day. In order to determine the four best images per food category for the diurnal LFPQ, the foods were validated by an online survey (*N* = 70 British participants, non-dieting, non-vegan, 67% female, 29 ± 15 years, completed the full survey). Respondents were asked to rate various food attributes on a visual analogue scale (VAS): “How pleasant does this food typically taste?” (0 = not at all, 100 = extremely); “Is this food more sweet or savory?” (0 = sweet, 100 = savory); “Is this food low or high in fat?” (0 = low, 100 = high); “How appropriate is it to consume this food in the morning (08:00–12:00 h)?” (0 = not at all, 100 = extremely); “How appropriate is it to consume this food in the afternoon (12:00–16:00 h)?” (0 = not at all, 100 = extremely); “How appropriate is it to consume this food in the evening (16:00–20:00 h)?” (0 = not at all, 100 = extremely). The resulting 16 foods to be used in the diurnal LFPQ, as well as their nutritional composition, are shown in Table 1. A 2 (AM or PM; within-subject factor) × 2 (savory or sweet; within-subject factor) × 2 (high or low fat; within-subject factor) repeated measures ANOVA showed there was no time-of-day interaction with taste and fat categories of food (*p* = 0.135). As a separate preliminary task, we internally piloted 10 potential test meals that could be consumed earlier and later in the day (i.e., as breakfast, lunch or dinner meals), and alongside the online validation survey results, the highest-rated test meal outcome was ‘beans on toast’. Accordingly, this was chosen as the test meal for the laboratory study.

### 2.2. Laboratory Study Design

After an initial session (see Section 2.4.1 for details), participants attended the laboratory on two separate occasions for early (AM; 8–10 a.m.) and late (PM; 4–6 p.m.) test sessions in a 2 × 2 counterbalanced design, 1 week apart. Chronotype grouping [early (EC) and late (LC) chronotype] was determined by Morningness–Eveningness Questionnaire (MEQ) score [15] median split, stratified for sex (calculated separately for each sex). Participants fasted for ≥3 h and refrained from exercise and alcohol for 24 h and caffeine for 12 h prior to each session. Compliance was confirmed verbally upon arrival for each test session. The test sessions assessed the subjective appetite and food reward response to a fixed-energy test meal (described in more detail below).

### 2.3. Participants

Fifty participants were recruited for the laboratory study and 44 completed the study (six withdrew due to scheduling issues). Participants were aged 18–25 years, non-vegetarian, non-breakfast skippers, not dieting or restricting food intake, and did not have a diagnosed eating disorder or any medical conditions that may be affected by the study requirements. This research was approved by the School of Psychology Research Ethics Committee at the University of Leeds (ref: PSC-550), and all participants provided written informed consent prior to taking part.

### 2.4. Measurements

#### 2.4.1. Initial Session

Participants attended an initial session where the MEQ [15] and Three-Factor Eating Questionnaire (TFEQ) [25] were used to assess chronotype and eating behavior traits, respectively. MEQ scores range between 16–86, with greater scores indicating more evening types. The TFEQ factors include dietary restraint, disinhibition and susceptibility to hunger, with higher scores indicating greater eating disturbances.

Following the questionnaires, anthropometrics (height, weight, and hip and waist circumference) and body composition via bioelectrical impedance (model BC-418, Tanita, UK) were measured. Percentage body fat was obtained from the Tanita output.

#### 2.4.2. Habitual Energy Intake

Habitual daily energy intake was assessed with an online self-administered 24-hour dietary record tool (myfood24, Leeds, UK) that has been previously validated against interviewer-based multiple pass recall and urinary biomarker concentrations [26]. However, both tools have similar degrees of underreporting relative to total daily energy expenditure estimated via a combination of indirect calorimetry and accelerometry (myfood24: −31%, multiple pass recall: −23%), highlighting limitations with dietary self-report tools. At the end of the initial sessions, participants were shown how to use the tool, which they completed on three non-consecutive days (two weekdays and one weekend day self-selected by the participants during the trial period). Participants were asked to report all foods and drinks consumed, keeping their food intake as habitual as possible. The nutrient and energy content of foods were calculated based on the McCance and Widdowson’s 6th Edition Composition of Foods UK Nutritional Dataset [27], supplemented with the nutrient content of fast food outlets and food packaging [28].

#### 2.4.3. Test Meal Sessions

Participants attended the laboratory between 8–10 a.m. for the AM session and between 4–6 p.m. for the PM session (all sessions were separated by 8 h across participants). A fixed energy test meal was composed of 195 g baked beans (Heinz) on 60 g toasted medium whole-meal bread (Sainsbury’s) with 300 mL ad libitum water (total meal = 300 kcal). Participants were given 15 min to consume the meal. Appetite ratings were assessed via pen and paper using 100-mm visual analogue scales (VAS) for hunger, fullness, desire to eat, prospective food consumption (PFC), appetite for something sweet and appetite for something savory [29]. Ratings were taken at seven time-points: baseline (−25 min), pre-meal (−15 min), post-meal (0 min), 15, 30, 45 and 60 min. A composite appetite score was calculated as the average of all the appetite ratings (fullness reversed) per time-point, as shown in Equation (1) [30].

Appetite = (Hunger + Desire + PFC + (100 − Fullness) + Appetite for sweet + Appetite for savory)/6(1)

After consumption of the test meal, additional ratings (100-mm VAS) assessed perceived test meal sweetness “How sweet did you find the meal?”, savoriness “How savory did you find the meal?”, fillingness “How filling did you find the meal?” and pleasantness “How pleasant did you find the meal?”, as well as prospective test meal consumption “How much more of this food do you think you could eat?” 

Food reward was assessed after the baseline and post-meal appetite VAS using the diurnal LFPQ to determine scores of implicit wanting and explicit liking for high-fat, low-fat, sweet and savory foods matched for familiarity, sweetness, protein, and acceptability [24], and as aforementioned in the validation section, for morning and evening appropriateness. Foods are categorized into high-fat savory, low-fat savory, high-fat sweet, and low-fat sweet (see Table 1). The LFPQ has been validated in a wide range of research [31,32,33]. Implicit wanting was assessed by asking the participants to select as fast as possible which of two foods from the four food categories “they most want to eat now”. Scores for implicit wanting were computed from mean response times adjusted for frequency. To measure explicit liking, the participants rated the extent to which they liked each food (“How pleasant would it be to taste this food now?”). The food images were presented individually in a randomized order and participants made their ratings using a 100-mm VAS. To calculate implicit wanting or explicit liking of fat appeal bias as a measure of hedonic preference for high-fat foods, low-fat food scores were subtracted from high-fat food scores, thus a positive score indicates greater implicit wanting/explicit liking for high-fat compared to low-fat foods [34].

### 2.5. Statistical Analyses

Data are presented as mean ± SD throughout the text and as mean ± SEM in the figures. Exact sample size from all available data for each analysis are reported in table and figure legends. Data were analyzed with SPSS (version 25, IBM, New York, NY, USA) and were normally distributed according to visual inspection. Pearson’s correlations were conducted to assess the relationships between variables. For the correlation analyses, liking and wanting variables were computed using the mean scores collapsed across all conditions (AM, PM, pre- and post-meal). Difference in participant characteristics between chronotype groups were assessed with independent samples t-tests. Appetite ratings were assessed with 2 (chronotype group; between-subject factor) × 2 (meal timing condition; within-subject factor) × 7 (time-points as described above; within-subject factor) repeated measures ANOVA. Food reward (pre-post meal scores collapsed) and test meal ratings were assessed with 2 (chronotype group; between-subject factor) × 2 (meal timing condition; within-subject factor) repeated measures ANOVA. Where appropriate, Greenhouse-Geisser probability levels were used to adjust for non-sphericity. Post hoc analyses were performed using the Bonferroni adjustment for multiple comparisons. Alongside *p*-values, estimated marginal mean differences (M_∆_) and 95% confidence intervals (95% CI) of the mean difference, as well as effect sizes as partial eta squared (ƞ_p_^2^) are reported. Typically, eta squared values are interpreted as small = 0.01, medium = 0.06 and large = 0.014 [35]; however, as there are no benchmark values for partial eta squared in the context of repeated measures designs [36], these should be interpreted with caution. Based on published data [37], a sample of 31 subjects (non-obese, both genders) should allow detection of a minimum difference of 7.5 mm in composite appetite ratings on a 100-mm VAS (≥ 80% power, alpha 0.05).

## 3. Results

### 3.1. Participant Characteristics 

Participant characteristics are shown in Table 2. There were eight males and 14 females within each chronotype group. By design, EC had an MEQ mean score greater than LC (*p* < 0.001; Table 2), but there were no other differences between chronotype groups. As reported previously in the literature, we found an inverse relationship between MEQ score and BMI (*r* = −0.370, *p* = 0.013), with EC showing a lower BMI. MEQ score was not associated with other anthropometric variables, TFEQ factors or habitual energy intake (all *p* ≥ 0.117).

### 3.2. Subjective Appetite Sensations by Meal Timing and Chronotype

**Figure 1:**
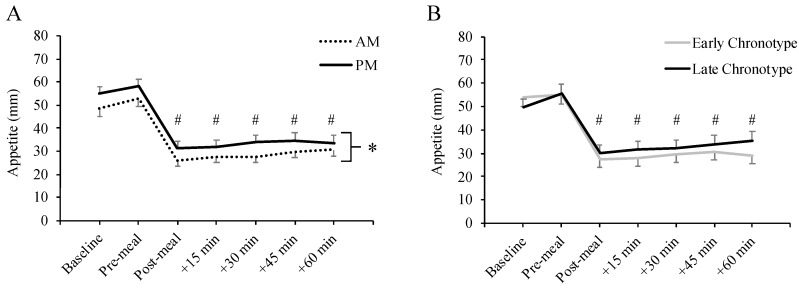
Composite appetite ratings according to meal timing condition (AM vs. PM; (**A**)) and chronotype group (Early vs. Late; (**B**)) * Main effect of meal timing condition, *p* = 0.040. ^#^ Different from baseline and pre-meal, *p* < 0.001. *N* = 39 (Early Chronotype *N* = 20, Late Chronotype *N* = 19). Data are mean ± SEM.

The overall mean appetite rating for AM EC was 34 (95% CI 27, 41) mm, AM LC was 35 (95% CI 29, 42) mm, PM EC was 38 (95% CI 31, 46) mm and PM LC was 41 (95% CI 33, 49) mm. As shown in Figure 1A, there was a main effect of meal timing on composite appetite score, with lower appetite in AM compared to PM (M_Δ_= −5 (95% CI −10, −0.2) mm, *p* = 0.040, η_p_^2^ = 0.109). There was also a main effect of timepoint (*p* < 0.001, η_p_^2^ = 0.648). There were no differences between chronotypes (Figure 1B; *p* = 0.630) or interactions among meal timing, chronotype and timepoint (*p* ≥ 0.250).

### 3.3. Test Meal Ratings by Meal Timing and Chronotype

**Figure 2:**
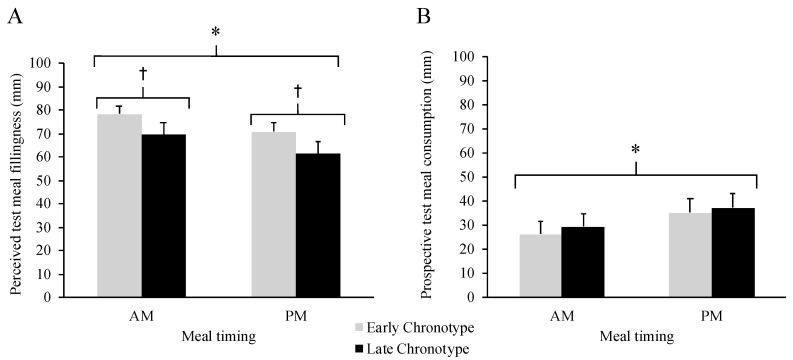
Ratings of perceived test meal fillingness (**A**) and prospective test meal consumption (**B**) according to meal timing condition (AM vs. PM) and chronotype (Early vs. Late). * Main effect of meal timing condition, *p* ≤ 0.017. ^†^ Main effect of chronotype group, *p* = 0.038. *N* = 41 (Early Chronotype *N* = 21, Late Chronotype *N* = 20). Data are mean ± SEM.

As shown in Figure 2A, perceived test meal fillingness was greater in AM compared to PM (M_Δ_= 8 (95% CI 1, 14) mm, *p* = 0.017, η_p_^2^ = 0.137) and in EC relative to LC (M_Δ_ = 9 (95% CI 0.5, 18], *p* = 0.038, η_p_^2^ = 0.105), but there was no interaction between meal timing and chronotype (*p* = 0.927). Prospective test meal consumption (Figure 2B) was lower in AM relative to PM (M_Δ_= −9 (95% CI −15, −2) mm, *p* = 0.011, η_p_^2^ = 0.154) but there were no differences between chronotypes and no interaction between meal timing and chronotype (*p* ≥ 0.722).

There were no differences in ratings of sweetness or pleasantness between the different meal timings and chronotypes, and no interactions between meal timing and chronotype (*p* ≥ 0.157). The test meal was perceived to be less savory in AM compared to PM (70 ± 18 vs. 77 ± 15 mm; *p* = 0.016, η_p_^2^ = 0.140), but there were no differences between chronotypes and no interaction between meal timing and chronotype (*p* ≥ 0.260).

### 3.4. Food Reward by Meal Timing and Chronotype

**Figure 3:**
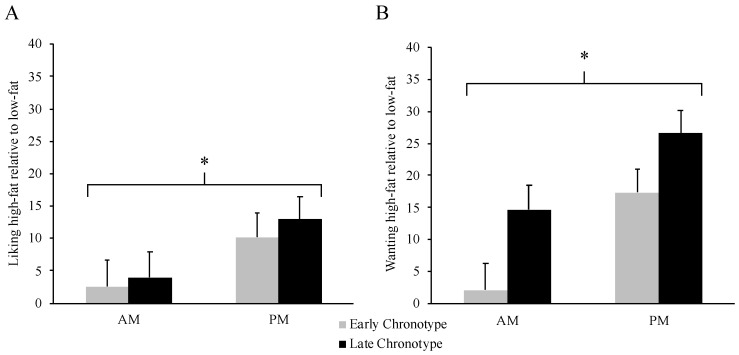
Liking (**A**) and wanting (**B**) scores (pre-post meal scores collapsed) for high-fat relative to low-fat foods according to meal timing condition (AM vs. PM) and chronotype (Early vs. Late). * Main effect of meal timing condition, *p* ≤ 0.004. *N* = 36 (Early Chronotype *N* = 17, Late Chronotype *N* = 19). Data are mean ± SEM.

As shown in Figure 3A, liking for high-fat relative to low fat foods was lower in AM relative to PM (M_Δ_= −8.3 (95% CI −13.7, −2.9], *p* = 0.004, η_p_^2^ = 0.222), but there were no differences between chronotypes (*p* = 0.661) or interaction between chronotype and meal timing (*p* = 0.812). As shown in Figure 3B, desire for high-fat relative to low fat foods was lower in AM relative to PM (M_Δ_= −13.6 (95% CI −20.8, −6.3], *p* = 0.001, η_p_^2^ = 0.299) with no differences between chronotypes (*p* = 0.208) or interaction between chronotype and meal timing (*p* = 0.658).

**Figure 4:**
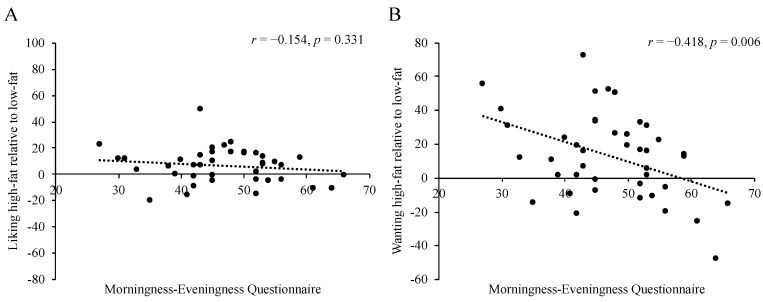
Relationship between scores of the Morningness–Eveningness Questionnaire and overall mean scores of liking (**A**) and wanting (**B**) for high-fat relative to low-fat food. Higher Morningness–Eveningness Questionnaire scores indicate earlier chronotypes, and positive liking/wanting scores indicate greater liking/desire for high-fat relative to low-fat. *N* = 42.

When MEQ score was correlated with mean scores of liking and wanting from all conditions (Figure 4), we found an inverse association between MEQ score and desire for high-fat food (*r* = −0.418, *p* = 0.006), such that EC had lower wanting. No relationship was found between MEQ score and liking for high-fat food. Wanting for high-fat food was also positively associated with total daily energy intake (*r* = 0.491, *p* = 0.002).

## 4. Discussion

With increasing evidence suggesting that meal timing may affect appetite and body weight control, experimental studies are required to assess outcomes and investigate underlying mechanisms using valid and reliable methodologies. The current study examined the impact of meal timing and chronotype under controlled laboratory conditions. We firstly validated a test meal and an array of food images that were time-of-day appropriate, then we assessed appetite and food reward responses in early (8–10 a.m.) and late (4–6 p.m.) meal timing sessions. Clear diurnal patterns of appetite and food reward—both lowest in the morning—as well as chronotype differences were observed, with the impact of meal timing and chronotype appearing to be an additive effect.

We found lower overall appetite in AM compared to PM, consistent with previous observational studies [13,14]. Forced desynchrony studies suggest the presence of an endogenous circadian rhythm in appetite, with greater evening hunger independent of wake time and energy intake [38,39]. This circadian pattern in appetite may stem from a greater metabolic response to meals in the morning (e.g., thermic effect of food, glucose tolerance, gastric emptying) [11,12], potentially promoting stronger satiety response [13,14]. Furthermore, there is evidence of circadian rhythms in acylated ghrelin secretion paralleling those of hunger [40], which may also explain the differences in appetite observed in the current study. Evidence regarding circadian patterns in satiety-related peptides such as glucagon-like peptide-1, peptide YY or cholecystokinin remains to be demonstrated. In the current study, there was no difference in the degree of appetite suppression between both meal timing conditions and chronotypes, suggesting a similar satiety response to food. Interestingly, the early chronotypes did appear to have slightly greater suppression of appetite in response to the test meal (exploratory post hoc t-test revealed a significant difference at 60 min post-meal, *p* < 0.05). It is possible that a larger meal (>300 kcal) might have shown differential effects on satiety responsiveness in the morning, as the energy content of the test meal used in the current study was more typical of a breakfast meal than an evening meal [13]. Nevertheless, overall appetite was lower in AM. Furthermore, test meal size was not calibrated according to BMI in this study, which may have improved the sensitivity of our design.

While appetite suppression in response to the test meal was similar across all conditions and groups, the test meal was rated more filling in AM compared to PM, and by early compared to late chronotypes. The lowest values observed were from the early chronotype in the AM condition, similar to their appetite ratings, suggesting an additive effect of meal timing and chronotype on perceived test meal fillingness. An uncoupling between subjective appetite response to food and measured satiety ratio (duration of after-meal interval divided by meal size from 7-day food diaries) has been previously reported in a large free-living sample (*n* = 867, age = 36 ± 14 years and BMI = 24.5 ± 4.3 kg/m^2^) [13,41]. Future studies utilizing more objective and controlled measures of satiety, such as a preload-test meal protocol, may help clarify these findings.

This is the first study to assess diurnal rhythms in food reward. We used the diurnal LFPQ with food image categories validated to be appropriate for early and late consumption to examine behavioral responses in explicit liking and implicit desire for high-fat relative to low-fat foods. Both liking and wanting scores were lower in AM relative to PM. Some small studies have examined diurnal and circadian rhythms of non-food reward in healthy young adults (with no information on weight status). One study assessed the influence of time-of-day on general liking and wanting, including taste–smell, among five other components, over seven free-living days using VAS on a smartphone software [42]. Peaks in liking and wanting were achieved at 6–7 p.m. Byrne & Murray [43] showed a peak in wanting (measured with the automatic Balloon Analogue Risk Task and International Affective Picture System arousal response) at 2 p.m. relative to 10 a.m. and 7 p.m. in males, while no diurnal variation in liking (measured with the International Affective Picture System pleasure response) was observed. Another study found time-of-day effects in neural (fMRI) response to a monetary reward task in the ventral striatum, with greater responses shown in later (~5 p.m.) relative to earlier (~10 a.m.) scans [44]. Lastly, a forced desynchrony study showed a circadian rhythm in positive affect and reward activation, operationalized as heart rate (calculated from ECG) and reaction time during the Fowles motor task (performed every 2–3 h per 28-h day), which coincided with core body temperature across circadian phases, peaking at 180°–240° (approx. 5–9 p.m.) [45]. While these non-food reward studies suggest circadian peaks in reward activation in the late afternoon/early evening, this may not directly compare to food reward and eating behaviors, as greater later evening and night-time eating has been associated with greater daily energy intake [13,14] and BMI [4,5,6]. Clearly, more work investigating extended time periods is needed to understand diurnal and circadian rhythms in food reward and their impact on food intake and susceptibility to overconsumption.

In terms of the impact of chronotype on food reward, in the whole sample, MEQ score was inversely associated with wanting but not liking. Interestingly, wanting scores were greatest in the late chronotypes in PM. While not directly comparable to the current study, a small study found that individuals with obesity and evening hyperphagia (consumption of ≥25% daily energy intake after evening meal) showed different mid-day pre- and post-meal neural response to food cues compared to matched-control participants [46]. This suggests that circadian eating patterns may be associated with altered diurnal neural responses to food. However, as that study only measured brain activity at one time-of-day in a specific sample, larger studies in populations with different cultural practices and objective measures of food intake are needed to clarify the role of food reward on food intake across the day, and if this is dependent upon chronotype.

We found an inverse association between MEQ score and BMI, similar to past studies showing earlier chronotypes associated with lower BMI [16,20]. However, unlike a previous larger study [20], we found no association between MEQ score and TFEQ factors, which may be due to our relatively smaller sample size. Furthermore, we found no association between MEQ score and self-reported daily energy intake from dietary record. However, as prior studies have found chronotype differences in daily energy intake patterns (i.e., proportion of energy intake in morning vs. evening) and not necessarily in daily energy intake [18], classifying energy intake patterns according to clock or circadian time may have been required to see differences in energy intake between chronotypes in the current study.

While this experimental study is one of the first to simultaneously assess effects of meal timing on appetite and food reward and associations with chronotype, several limitations should be acknowledged and addressed in future research. While participants were requested to fast ≥3 h prior to testing, some participants may have incurred a longer fasting period in the early meal timing condition than the late meal timing condition (i.e., if they fasted overnight), but unfortunately the actual duration of the fasting period was not measured. MEQ scores in our sample shifted relatively toward the morning/intermediate range of the scale and the median split applied to the data did not result in an evening chronotype group comparable to the definition proposed by the original authors (i.e., scores < 41 as indicative of ‘evening types’) [14]. Meal timing was based on clock time and not individual circadian time. Menstrual cycle was not controlled for, which may have influenced the results. Finally, this study was conducted in young British adults with a relatively healthy BMI, which limits the generalization of the findings and of the specific foods validated for this methodology. Future studies outside of the UK should culturally adapt and validate food images for the diurnal assessment of food reward using the Leeds Food Preference Questionnaire in their specific population [34].

## 5. Conclusions

We examined the impact of meal timing and chronotype with a validated, time-of-day appropriate test meal and array of images to assess food reward. Meal timing and chronotype appeared to have an additive rather than interactive effect on perceived satiety and food reward. Early meal timing was associated with lower appetite, greater test meal perceived fillingness and lower liking and desire for high-fat food, whereas early chronotype was associated with lower BMI, greater test meal perceived fillingness, and lower wanting but not liking for high-fat food. These data suggest that meal timing and chronotype should be considered in the assessment and interpretation of appetite and food reward outcomes.

## Figures and Tables

**Table 1:** Validation data of diurnal LFPQ food images.

	Food	AM Appropr. Mean ± SD	PM Appropr. Mean ± SD	E% Protein	E% CHO	E% Fat	Energy Density (kcal/100 g)
High-Fat Savory	Cashews	46 ± 32	75 ± 18	13.7	13.8	70.2	642
	Croissant, ham & cheese	79 ± 24	51 ± 23	17.3	25.3	54.9	345
	Sausage roll	51 ± 33	67 ± 20	10.4	22.3	64.9	361
	Sausage sandwich	80 ± 24	62 ± 23	24.4	22.8	50.1	279
	Mean	65	62	16.5	21.1	60.0	407
Low-Fat Savory	Beans on toast	81 ± 22	74 ± 21	19.1	66.5	4.6	122
	Bread roll	76 ± 26	80 ± 18	13.2	69.8	9.8	258
	Rice cake	57 ± 32	67 ± 23	8.5	77.0	6.5	386
	Rye crackers (plain)	61 ± 34	67 ± 23	9.7	71.7	4.4	350
	Mean	68	71	12.6	71.2	6.3	279
High-Fat Sweet	Blueberry muffin	59 ± 31	64 ± 22	5.5	44.1	46.4	367
	Cinnamon swirl pastry	69 ± 27	53 ±25	4.9	37.7	53.8	445
	Crepe with cream	76 ± 28	59 ± 24	4.6	16.1	79.3	263
	Flapjack	57 ± 29	69 ± 21	4.9	48.3	41.4	435
	Mean	65	60	5.0	36.6	55.2	377
Low-Fat Sweet	Dried apricots	75 ± 27	73 ± 21	8.8	75.8	3.0	178
	Red grapes	82 ± 24	86 ± 17	2.4	87.5	1.4	66
	Banana	91 ± 13	82 ± 20	4.7	83.7	4.4	103
	Light chocolate granola bar	83 ± 19	60 ± 22	5.8	61.1	16.2	344
	Mean	83	75	5.4	77.0	6.3	173

Average appropriateness score for consumption in morning (AM appropr.); carbohydrate (CHO); percentage of total daily energy intake (E%), average appropriateness score for consumption in afternoon/evening (PM appropr.). *N* = 70.

**Table 2:** Participant characteristics.

	All (*N* = 44)	Early Chronotype (*N* = 22)	Late Chronotype (*N* = 22)
Weight (kg)	72.9 ± 11.4	73.4 ± 10.3	72.4 ± 12.7
BMI (kg/m^2^)	24.5 ± 3.2	24.1 ± 2.7	24.9 ± 3.6
Body fat (%)	27.7 ± 8.3	27.3 ± 8.4	28.2 ± 8.4
Hip circumference (cm)	98.4 ± 6.9	99.2 ± 4.8	97.6 ± 8.6
Waist circumference (cm)	84.3 ± 7.9	84.2 ± 6.2	84.3 ± 9.4
Waist-to-hip ratio	0.86 ± 0.06	0.85 ± 0.07	0.86 ± 0.06
MEQ score	48 ± 9	55 ± 5	40 ± 6 *
TFEQ Restraint	7 ± 4	6 ± 3	8 ± 5
TFEQ Disinhibition	8 ± 3	7 ± 3	8 ± 3
TFEQ Hunger	7 ± 3	7 ± 3	7 ± 3
Energy intake (kcal/day) ^1^	1791 ± 663	1843 ± 681	1737 ± 659

Morningness–Eveningness Questionnaire (MEQ); Three-Factor Eating Questionnaire (TFEQ). Data are mean ± SD; ^1^ *N* = 37 (Early Chronotype *N* = 19, Late Chronotype *N* = 18). * Early vs. Late *p* < 0.001.
